# Anti-diabetic Effect of *Punica granatum* Flower Polyphenols Extract in Type 2 Diabetic Rats: Activation of Akt/GSK-3β and Inhibition of IRE1α-XBP1 Pathways

**DOI:** 10.3389/fendo.2018.00586

**Published:** 2018-10-15

**Authors:** Dan Tang, Liu Liu, Dildar Ajiakber, Jianping Ye, Jianjun Xu, Xuelei Xin, Haji Akber Aisa

**Affiliations:** ^1^State Key Laboratory Basis of Xinjiang Indigenous Medicinal Plants Resource Utilization, Xinjiang Technical Institute of Physics and Chemistry, Chinese Academy of Sciences, Urumqi, China; ^2^University of Chinese Academy of Sciences, Beijing, China; ^3^Shanghai Institute of Materia Medica, Chinese Academy of Sciences, Shanghai, China; ^4^Pennington Biomedical Research Center, Louisisana State University, Baton Rouge, LA, United States

**Keywords:** type 2 diabetes mellitus, *Punica granatum* L. flower, anti-diabetic herb, oral glucose tolerance test, endoplasmic reticulum stress, insulin resistance

## Abstract

Type 2 diabetes mellitus (T2DM) is the most common type of diabetes with more than hundreds of millions of patients worldwide. However, the medicines for treatment of T2DM are very limited. In China, *Punica granatum* L. flower (PGF) has been used as an anti-diabetic herb in the herbal medicine. The activity involves in improvement of insulin sensitivity. However, the underlying mechanism of action is elusive. The current study was designed to address this issue by investigating the effect of polyphenols extract of PGF in diabetic rats. A rat model was orally administrated with PGF polyphenols extract at doses of 50 and 100 mg/kg for 4 weeks. Insulin sensitivity was improved as indicated by oral glucose tolerance test (OGTT), insulin tolerance test (ITT) and homeostasis model assessment of insulin resistance (HOMA-IR). At the molecular level, insulin signaling activity was improved with an elevation in insulin-stimulated phosphorylation of insulin receptor substrate (IRS-1), Akt and GSK-3β. Endoplasmic reticulum (ER) stress signals including phosphorylation of inositol-requiring kinase1 (IRE1) and activation of X box binding protein (XBP-1) splicing were decreased by the PGF treatment. Expressions of IRE1α, XBPs, and CHOP were all decreased by PGF. Blood lipid profile, liver glycogen content and antioxidant status were improved by PGF in the rats. The observations suggest that PGF is able to lower glucose levels in T2DM rats by improving the insulin resistance. The mechanism is likely related to the activation of Akt-GSK3β signaling pathway and inhibition of ER stress.

## Introduction

Diabetes mellitus (DM) is one of the most common chronic metabolic diseases throughout the world. Type 2 diabetes mellitus (T2DM) accounts for about 90% of diabetic cases, and exhibits a characteristic of insulin resistance, which is observed as hyperglycemia in the presence of hyperinsulinemia. Diabetes threatens human life through serious diabetic complications including retinopathy, nephropathy and autonomic neuropathy (1, 2). Currently synthetic anti-diabetic drugs are widely used in the treatment of type 2 diabetes, but the side effects limit application of the medicines (3). It is generally believed that the traditional herbal medicines have an advantage in the prevention and treatment of diabetes for less side effects (4). Identification of the bioactive components is a major interest in the study of herbal medicines.

*Punica granatum* Linn. (Pomegranate) is a medical and edible plant in the southeast Asia, the Mediterranean and the northwest region of China. *Punica granatum* flowers (PGF) is prescribed in Unani and Ayurvedic medicines in the treatment of diabetes for centuries (5, 6). In China, the traditional Uygur medicine use the flowers of pomegranate as a remedy for diabetes (7). PGF extract contains a large amount of polyphenols and possesses potent anti-inflammatory and antioxidant effects (8, 9). PGF was reported to reduce blood glucose, lipid and ameliorate insulin resistance in diabetic animal models (10–13). PGF exhibited antihyperglycemic activity by activiating peroxisome proliferator-activated receptor gamma (PPAR gamma), which is a potential target affected by PGF (14–16). Although pomegranate flower has been reported in many studies for its anti-diabetic activities, the studies on molecular mechanisms of PGF for are relatively limited.

Endoplasmic reticulum (ER) stress has been implicated in causing hepatic insulin resistance. ER stress contributes to glucose disorder in type 2 diabetes by induction of insulin resistance or inhibition of secretion of insulin (17). Under the condition of diabetes, endoplasmic reticulum (ER) stress are induced, triggering the activation of unfolded protein response (UPR) (18, 19). Activation of inositol-requiring enzyme 1 (IRE1) by ER stress is a critical step in the initiation of UPR signaling branches (20, 21). Following IRE1α activation, the transcription factor X-box binding protein 1 (XBP1) is transformed from the inactive form to the active form (XBP-1s) by non-conventional splicing (22). The IRE1-XBP1 pathway contributes to the pathogenesis of insulin resistance through serine phosphorylation of insulin receptor substrate 1(IRS-1) (23). The pathway also involves in inhibition of insulin secretion in pancreatic islet β-cells (24, 25). Over-expression of IRE1α promotes degradation of proinsulin mRNA to inhibit insulin secretion (26). Herein, we want to known if the pathway is regulated by PGF in the improvement of insulin sensitivity.

In our previous studies, 14 chemical compositions in PGF were identified or partially characterized by LC/QTOF and MS/MS, which include flavonoids and tannins compounds (ellagic acid, gallic acid, granatin, luteolin, and quercetin). The polyphenol compounds in PGF mentioned above possessed potent inhibitory activity against α-glucosidase suggesting anti-diabetic potential (27). In this study, we further investigated the effects of PGF on a streptozotocin/high-fat-diet (STZ/HFD)-induced T2DM rat model. The impact of PGF on insulin signaling pathway and IRE1-XBP1 pathway was examined. We aimed to elucidate the mechanisms of action for the anti-diabetic effect of PGF.

## Materials and methods

### Materials and reagents

Streptozotocin (STZ) was purchased from Sigma Chemical (USA). Metformin (MET, positive drug) was purchased from Sino-US Shanghai Squibb Pharmaceutical Co., Ltd (China). Human recombinant insulin was obtained from Sigma Chemical Corp. (St. Louis, MO, USA). Rat insulin ELISA kit was purchased from EMD Millipore Corporation, USA. Kits of catalase (CAT), Superoxide Dismutase (SOD), lipid peroxidation (MDA), and glutathione peroxidase (GSH) were all purchased from Nanjing Jiancheng Bioengineering institute (NJJCBIO,China). Electrophoresis reagents, including Bis-Tris gels, running buffer, and polyvinylidene fluoride (PVDF) membrane were obtained from Invitrogen (Carlsbad, CA, USA). Antibodies to XBP1, CHOP, IRE1α, eIF2α, p-eIF2α, and β-actin, phospho-Akt (p-Akt), phospho-GSK-3β (p-GSK-3β), phospho-IRS-1 (p-IRS-1(try)), Akt, GSK-3β and IRS-1 and the secondary antibody were obtained from the Cell Signaling Technology (Danvers, MA, USA). The p-IRE1α (S724) antibody was obtained from Abcam (Cambridge, MA, USA). Normal diet/standard chow (Research Diets #D12450B, 10% calorie in fat, energy density 3.85 kcal/g) and high-fat diet (Research Diets #D12492, 60% calorie in fat, energy density 5.24 kcal/g) were ordered from Research Diets, Inc. (New Brunswick, NJ). Lysis buffer was prepared with 25 mM HEPES pH 7.8, 50 mM KCl, 1% NP-40, 10 μg/ml leupeptin, 20 μg/ml aprotinin,125 μM DTT, 1 mM Na_3_VO_4_, and 1 mM PMSF (pH 7.5).

### Preparation of PGF and HPLC-QTOF-MS/MS analysis

The *Punica granatum* flowers (PGF) were collected in the hotan district of Xinjiang Uygur Autonomous Region, China, and the identity was proved by Prof. Guanmian Shen (Xinjiang Institute of Ecology and Geography, Chinese Academy of Sciences). The voucher specimens of PGF are deposited in Herbarium, Xinjiang Institute of Ecology and Geography and its herbarium number is 00022757. The optimal extraction and purification processes for the pomegranate flower polyphenols were carried out according to a protocol in our published study (28, 29). The dried pomegranate flower was extracted with ethanol under reflux three times. After removal of ethanol with rotary evaporator under vacuum conditions at 45°C, the residual was subjected to a D-101 macroporous resin glass chromatography column with a 1:4 diameter height ratio. The desired fractions were eluted with 70% (v/v) ethanol and concentrated in a vacuum condition as PGF extract. The characterisation of compounds in PGF extract was analyzed by high performance liquid chromatography coupled to quadruple time of-flight with tandem mass spectrometry (HPLC-QTOF-MS/MS).

### Animals and experimental protocol

Male Sprague-Dawley rats (SD rats) of 6 weeks in age (weighting 200 ± 20 g) were obtained from and maintained in the Center of Laboratory Animals of Xinjiang Medical University (Urumqi, P. R. China). The animals were housed in standard polypropylene cages in an air-conditioned room with a 12 h day-night cycle, temperature of 24 ± 3°C and relative humidity of 45–65%. All experiments were approved (Protocol Approval No.2015006) by the Animal Care and Use Committee (IACUC) of the Xinjiang Medical University.

Animals were supplied with fresh water and food *ad libitum* for 1 week before the induction of experimental model. The rats were randomly divided into four groups: one group was given the normal diet (control group); the other three groups were fed on a high-fat diet (HFD groups). After 6 weeks on HFD, the rats were fasted overnight and injected with a low dose of STZ (30 mg/kg body weight) except the rats in the control group. STZ solution was freshly prepared in a dark condition at 0.1 mmol/L in a cold citrate buffer (pH 4.4) (30). One-week after the STZ injection, the fasting blood glucose (FBG) levels were measured in the rats using the glucometer (ACCU-CHEK, ROCHE). Rats with FBG above 11.1 mmol/L were used as diabetic rats (31). The animals were divided into four groups (*n* = 10/group) as follows:

1. Normal control group (NC): healthy rats were orally treated with saline
2. Diabetes model group (DM): HFD-STZ induced diabetic rats were orally treated with the same volume of saline
3. Metformin-treated DM group (DM+MET): DM rats were given metformin at 150 mg/kg by gavage for 4 weeks
4. PGF-treated DM groups (DM+PGF): DM rats were treated with PGF at low and high dosages (50 and 100 mg/kg, respectively) by oral gavage for 4 weeks

NC group was fed on chow diet throughout the study. The model and treatment groups were fed on HFD in the study. Food and water intake were recorded once a week. Fasting blood glucose and body weight were measured weekly. Oral glucose tolerance test (OGTT) and insulin tolerance test (ITT) were performed in the last week of PGF treatment. After 4-weeks pharmacological intervention, the rats were fasted for 12 h and sacrificed for tissue collection. The plasma and tissue samples were collected and stored at −80°C for subsequent assays.

### Measurement of fasting blood glucose

The fasting blood glucose (FBG) was measured weekly in the rats during the treatment. Blood samples were collected from the rat tail vein, and measured using a Roche glucometer according to the manufacturer's instructions. The results are expressed as mmol/L plasma.

### Glucose and insulin tolerance tests

Oral glucose tolerance test (OGTT) was performed after 12 h overnight fasting. The rats were given glucose (2 g/kg body weight) by gavage of 40% solution and the blood samples were collected from the tail vein at 0, 30, 60, and 120 min for the blood glucose using the Roche glucometer. The area under curve (AUC) was calculated to represent the glucose tolerance. For insulin tolerance test (ITT), after 4 h of fasting, rats were given an intraperitoneal injection of 0.75 U/kg insulin. Glucose level was measured immediately with a glucometer at 0, 30, 60, and 120 min. The value is presented as a percentage of initial plasma glucose level. The areas under the curve (AUC) were calculated to represent the insulin tolerance. AUC (mmol/L·min) was calculated using the formula AUC_glucose_ = 0.5 × (G_0_+G_30_)/2 + 0.5 × (G_30_+G_60_)/2 + 1 × (G_60_ + G_120_)/2, where G_0_, G_30_, G_60_, G_90_, and G_120_ were blood glucose values at different time points (32).

### Blood biochemistry and antioxidant enzyme activities

After overnight fasting, blood was collected from the rats at the orbital venous sinus. Plasma was isolated by centrifuge at 3,500 × g for 10 min. The fasting blood glucose (FBG), total cholesterol (TC), triglycerides (TG) and high-density lipoprotein cholesterol (HDL-C) were determined in the plasma using an automatic biochemical analyzer (Roche-Cobas Integra 400 Plus). Fasting Insulin levels (FINS) were quantified using the ELISA kit, which was obtained from Millipore Corp. (Bedford, MA, USA). In addition, free fatty acid (FFA), glycogen content and hexokinas were determined in the rat liver using the corresponding assay kits (NJJCBIO, China) according to the manufacturer's instructions. Glutathione peroxidase activity (GSH-Px), superoxide dismutase activity (SOD), catalase (CAT) activity, and malondialdehyde (MDA) were measured in the liver using the commercial kits (NJJCBIO, China).

### Homeostasis model assessment of insulin resistance and β-cell function

Homeostasis model assessment of insulin resistance (HOMA-IR) and β-cell function (HOMA-β) were calculated with the fasting blood glucose (FBG) and insulin values (FINS) in the evaluation of the degree of insulin resistance and islet β-cell function. Insulin sensitivity index [ISI] reflects on the efficiency of insulin in response to glucose intake in the body. [HOMA-IR] = FBG (mmol/l) × FINS (mU/ml)/22.5 (33, 34). [HOMA-β] = 20 × FINS/(FBG-3.5) (35). ISI = Ln [(FBG × FINS)^-1^] (36, 37).

### Western blotting

The liver tissue were dissolved in ice-cold lysis buffer (1:10), and homogenized using QIAGEN Tissuelyser LT (5 min, 50 Hz) followed by centrifugation at 13,000 rpm for 10 min at 4°C. Protein concentration was determined using the BCA assay (Thermo Scientific, Rockford, IL, USA). The proteins were resolved in 8% SDS-PAGE and transferred to PVDF membranes. The PVDF membrane was blocked in a 5% BSA-TBST buffer for 1 h at room temperature, then incubated with the primary antibody (1:1,000) at 4°C overnight. After washing three times, the membrane was incubated with the secondary antibody for 1 h at room temperature. The protein signals were detected in the membrane using the ECL Plus chemiluminescent reagent (GE Heathcare UK Ltd., Little Chalfont, Buckinghamshire, UK). The protein signals of interest were collected using the Bio-Rad ChemiDoc™ XRS imaging system.

### RNA preparation and RT- PCR

Total RNA was extracted from the liver tissues using TRIzol reagent (Ambion®, Life Technologies) according to the manufacturer's instruction. The RNA concentration was determined by measuring absorbance at 260 nm using a NanoDrop 2000c spectrophotometer (Thermo Scientific, Waltham, MS, USA). RNA samples were stored at −80°C until the test.

RT-PCR was performed in the evaluation of mRNA expression. mRNA was reverse transcribed into the first-strand cDNA using the High Capacity cDNA Reverse Transcription Kit (Thermo Fisher Scientific). One-tenth of resulting cDNA was used as template for mRNA quantification by PCR. The resulting PCR products were analyzed by electrophoresis on a 1% agarose gel containing SYBR green and the image was taken using the Bio-Rad imaging system. GAPDH was used as an endogenous control. The PCR results were expressed as the relative expression ratio (between targeted gene and internal control GAPDH). All the PCR primers were obtained from Invitrogen Corporation (Carlsbad, California, USA). The primers in this study were followings: IRE1α, forward 5′-GCG AAC AGA ATA CAC CAT CAC-3′, reverse 5′-ACC AGC CCA TCA CCA TTG-3′; XBPs, forward 5′-TGC TGA GTC CGC AGC AGG TG-3′, reverse 5′-GCT GGC AGG CTC TGG GGA AG-3′; PERK, forward 5′-GAA CCA GAC GAT GAG ACA GAG-3′, reverse 5′-GGA TGA CAC CAA GGA ACC G-3′; CHOP, forward 5′-GTA CCT ATG TTT CAC CTC CTG G-3′, reverse 5′-TGG AAT CTG GAG AGT GAG GG-3′; ATF-6, forward 5′-CCT GTC CTA CAA AGT ACC ATG AG-3′, reverse 5′-CCT TTA ATC TCG CCT CTA ACC C-3′; GAPDH, forward 5′-AAG AAG GTG GTG AAG CAG GC-3′, reverse 5′-TCC ACC ACC CTG TTG CTG TA-3′.

### Statistical analysis

All results were expressed as mean ± *SD*. Data were analyzed by one-way analysis of variance (ANOVA). SPSS version 19.0 (SPSS Inc., Chicago, IL, USA) was used to perform all of the statistical analysis. *P*-values less than 0.05 (P < 0.05) were considered as indicative of significance.

## Results

### Reduction of weight gain by PGF

Energy surplus such as weight gain is a major cause of insulin resistance in the pathogenesis of T2DM (38). To understand PGF effect in the regulation of insulin sensitivity, energy balance was examined in PGF treated rats by monitoring the body weight, food and water intake during the 4-weeks study (Table 1). The rats on HFD (DM) gained more body weight (^*##*^*p* < 0.05) than the rats on chow diet (NC). However, the weight gain was significantly decreased (^*^p < 0.05) by PGF at the two dosages tested (50 and 100 mg/kg). The weight reduction was also observed in rats treated with metformin (MET, 150 mg/kg). The PGF effect was not a result of inhibition of food intake, which was not changed by the treatment. The DM rats had a 19.3% higher (^##^*p* < 0.01) water consumption than NC rats due to the diabetes status. The increase in water intake was reversed by PGF treatment at the two dosages as well as by MET. The results suggest that PGF treatment was able to attenuate the weight gain and correct the increase in water intake in DM rats.

**Table 1:** Body weights, food and water intake among the five groups (Mean ± *SD, n* = 10).

**Group**	**NC**	**DM**	**DM+MET**	**DM+PFL**	**DM+PFH**
Initial BW(g)	509.88 ± 22.5	509.15 ± 27.1	506.95 ± 24.1	509.61 ± 29.3	508.8 ± 25.9
Final BW (g)	556.18 ± 29.5	581.66 ± 34.2^#^	555.22 ± 42.3	555.66 ± 39.2^*^	558.0 ± 42.2^*^
BW gain (g)	46.30 ± 8.9	72.51 ± 19.2^#^	48.27 ± 17.5	47.05 ± 11.3^*^	49.20 ± 9.50^*^
Food intake\ (g/kg BW/day)	28.25 ± 4.15	27.5 ± 2.63	25.14 ± 4.63	23.6 ± 4.61	25.2 ± 4.66
Water intake (mL/kg BW/day)	59.63 ± 6.07	68.1 ± 8.09^#^	58.63 ± 8.63^*^	54.73 ± 12.65^*^	58.6 ± 12.42^**^

NC, Normal control group; DM, Diabetic rat group; DM + MET, Metformin treatment group; DM + PFL, Low-dose PGF treatment group; DM + PFH, High-dose PGF treatment group; BW, Body weight; Data are expressed as mean ± SD (n = 10).

# p < 0.05,

## p < 0.01 for significant difference from the NC group.

* p < 0.05,

** *p < 0.01 for significant difference from the DM group. PFL: 50 mg/kg, PFH: 100 mg/kg. MET: 150 mg/kg*.

### Effect of PGF on fasting blood glucose

**Figure 1:**
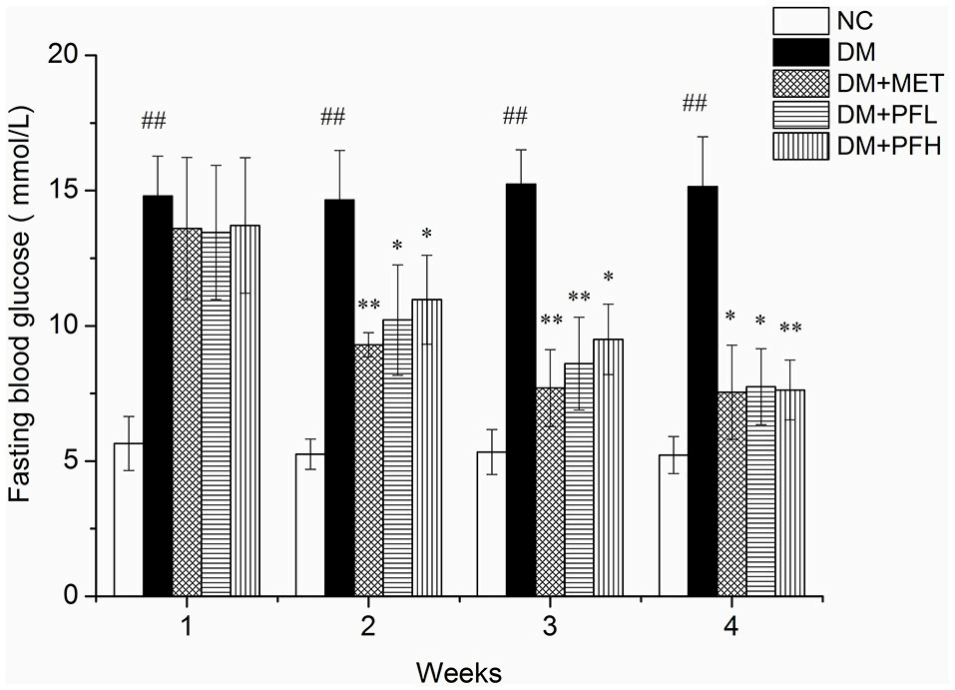
Effects of PGF in diabetic rats on fasting plasma glucose during the 4 weeks of treatment. The DM rats induced by streptozotocin (STZ) were orally treated with PGF at low (50 mg/kg) and high doses (100 mg/kg), or metformin (150 mg/kg) for 4 weeks. In the 4th week, PGF obviously reduced the fasting plasma glucose, exhibiting a same efficacy to metformin in the control of glucose level. Data set shown is representative of three experiments. Data are represented as as mean ± *SD* (*n* = 10/group). ^##^*P* < 0.01, ^#^*P* < 0.05 vs. NC; ^**^P < 0.01, ^*^P < 0.05 vs. DM.

Elevation of fasting blood glucose (FBG) is an indicator of insulin resistance in T2DM. To test the PGF effect on insulin resistance, blood glucose was measured in the rats weekly during the PGF treatment. As shown in Figure 1, FBG level was increased in the DM group (^*##*^*p* < 0.01) over the NC group during the 4 weeks study. The elevation was reduced by PGF in a time-dependent manner in the DM rats. In the first week, FBG was not significantly altered by PGF. In the second week, FBG was reduced by PGF, but the level was still well above the normal (>10 mmol/l) (^*^p < 0.05). In the 4th week, a significant decrease in FBG was observed in PFL (49%, ^*^p < 0.05) and PFH (50%, ^**^p < 0.01) groups compared with DM group. There was no obvious difference in FBG levels among PFL, PFH and metformin groups, suggesting that PGF exhibited a same efficacy to metformin in the control of insulin resistance.

### Effect of PGF on OGTT and ITT

**Figure 2:**
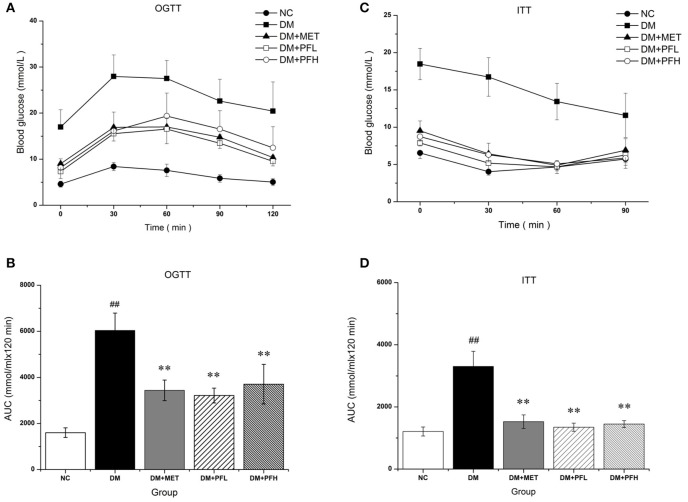
The effect of PGF on glucose metabolism *in vivo*. **(A)** For OGTT, following an overnight fast, the rats were orally injected of glucose at 2 g kg−1 BW. Blood glucose level was measured in tail vein blood at 0, 30, 60, 90, and 120 min. **(B)** Area under the curve (AUC) data was calculated for OGTT. Impaired glucose tolerance was improved by PGF. **(C)** For ITT, following a 4 h fasting, the rats were injected intraperitoneally of insulin at 0.75 U kg−1 BW. Blood glucose levels were measured at 0, 30, 60, and 120 min. **(D)** AUC data were calculated for ITT. The insulin sensitivity are increased by PGF. Data set shown is representative of three experiments. Data are represented as as mean ± *SD* (*n* = 6 /group). ^##^*P* < 0.01, ^#^*P* < 0.05 vs. NC; ^**^P < 0.01, ^*^P < 0.05 vs. DM.

OGTT was performed to assess the ability of the animals to dispose a glucose challenge. Diabetic rats showed statistically elevated blood glucose levels compared to normal control rats before and after glucose load (Figure 2A). The increased glucose area under the curve (AUC) on the OGTT in DM group indicated severe glucose intolerance in diabetic rats (^##^*p* < 0.01, Figure 2B). Diabetic rats treated with PFL and PFH exhibited a reduced glucose AUC by 47 and 38% (^**^P < 0.01). The results suggest PGF at both doses significantly improved the impaired glucose tolerance, and the low dose (PFL) group appears to have better effect. In addition, the efficacy of PGF was comparable to that of metformin.

After insulin injection, blood glucose of normal rats drops rapidly during the 30 min, while blood glucose levels in DM group drops slowly during 2 h (Figure 2C). DM group showed a significantly different glucose response curve with a larger AUC compared to the other four groups (Figure 2D). Diabetic rats displayed a delayed response to exogenous insulin, suggesting a impairment of insulin tolerance in DM group. The impaired insulin tolerance was improved by 61 and 58% with PFL (^**^p < 0.01) and PFH (^*^p < 0.05) as indicated by AUC. The data suggest that PGF improved insulin sensitivity in diabetic rats.

### Effect of PGF on serum glucose and insulin levels

Efficacy of PGF in the improvement of insulin sensitivity was further tested with HOMA-IR index, which is calculated with a formula in the basis of fasting blood glucose (FBG) and fasting insulin (FINS). The index was 4 times higher in DM rats over NC rats (^##^*p* < 0.01) for the insulin resistance (Table 2). The index was significantly reduced by PGF at the low and high levels (^*^p < 0.05). The efficacy of PGF was comparable to that of metformin. There was no significant difference between PGF and metformin groups in the improvement of HOMA-IR index. Insulin sensitivity index (ISI) was used to assess the status of insulin sensitivity. Compared with NC group, ISI decreased obviously in DM group (^#^*p* < 0.05). The decrease was corrected by PFL and PFH (^*^p < 0.05). We found no significant difference among PFL, PFH and metform groups. The data suggest that insulin sensitivity was improved by PGF in the diabetic rats with an efficacy identical to that of metformin.

**Table 2:** FBG, FINS, ISI, HOMA-IR and HOMA-β in rats serum of five groups (Mean ± *SD, n* = 10).

**Groups**	**FBG (mmol/L)**	**FINS (mIU/L)**	**ISI**	**HOMA-IR**	**HOMA-β**
NC	6.03 ± 0.27	18.19 ± 3.39	−4.69 ± 0.32	4.88 ± 0.79	143.79 ± 27.78
DM	16.40 ± 5.79^##^	30.09 ± 4.24^##^	−6.21 ± 0.76^#^	21.94 ± 3.31^##^	46.65 ± 5.22^##^
DM+PFL	8.83 ± 1.11^**^	19.51 ± 1.99^*^	−5.14 ± 0.27^*^	7.65 ± 0.65^*^	73.21 ± 15.61
DM+PFH	8.35 ± 0.71^**^	18.91 ± 2.51^*^	−5.06 ± 0.41^*^	7.02 ± 0.77^*^	77.97 ± 16.37
DM+MET	8.78 ± 0.85^*^	19.37 ± 3.69^*^	−5.13 ± 0.52^*^	7.55 ± 0.89	73.37 ± 13.14

FBG, Fasting blood glucose; FINS, Fasting insulin; ISI, Insulin sensitivity index; HOMA-IR, Insulin resistance index; HOMA-β: β-cell insulin secretion function index. Data are expressed as mean ± SD (n = 10).

# p < 0.05,

## p < 0.01 for significant difference from the NC group.

* p < 0.05,

** *p < 0.01 for significant difference from the DM group. PFL: 50 mg/kg, PFH: 100 mg/kg. MET: 150 mg/kg*.

### Effect of PGF on serum lipid profiles

Parameters of blood lipids were examined in this study, including triglyceride (TG), total cholesterol (TC), free fatty acids (FFA), and high density lipoprotein C (HDL-C). A significant increase was observed in DM rats in the levels of TG, TC, FFA, and HDL-C. However, the increases were significantly reduced by PGF at the low and high dosage (PFL, ^*^p < 0.05 and PFH, ^**^p < 0.01) (Table 3). The efficacy of PGF was similar to that of metformin in the inhibition of blood lipids.

**Table 3:** FFA, TC, TG and HDLc in rat serum (Mean ± *SD, n* = 10).

**Groups**	**TG (mmol/L)**	**TC (mmol/L)**	**HDL-C (mmol/L)**	**FFA (μmol/L)**
NC	0.64 ± 0.12	1.69 ± 0.13	1.22 ± 0.22	325.20 ± 53.55
DM	1.62 ± 0.74^#^	2.85 ± 0.23^##^	0.75 ± 0.15^#^	579.22 ± 74.86^##^
DM+PFL	0.73 ± 0.18^*^	1.74 ± 0.21^*^	1.19 ± 0.13^*^	355.42 ± 73.75^*^
DM+PFH	0.65 ± 0.13^**^	1.68 ± 0.19^**^	1.12 ± 0.11^*^	288.64 ± 60.40^**^
DM+MET	0.68 ± 0.12^*^	1.74 ± 0.18^*^	1.16 ± 0.09^*^	278.82 ± 74.38^*^

TG, Triglycerides; TC, Total cholesterol; HDL-C, High-density lipoprotein cholesterol; FFA, Free fatty acid. Data are expressed as mean ± SD (n = 8).

# p < 0.05,

## p < 0.01 for significant difference from the NC group.

* p < 0.05,

** *p < 0.01 for significant difference from the DM group. PFL: 50 mg/kg, PFH:100 mg/kg, MET:150 mg/kg*.

### Effect of PGF on glycogen content and antioxidant enzymes

**Figure 3:**
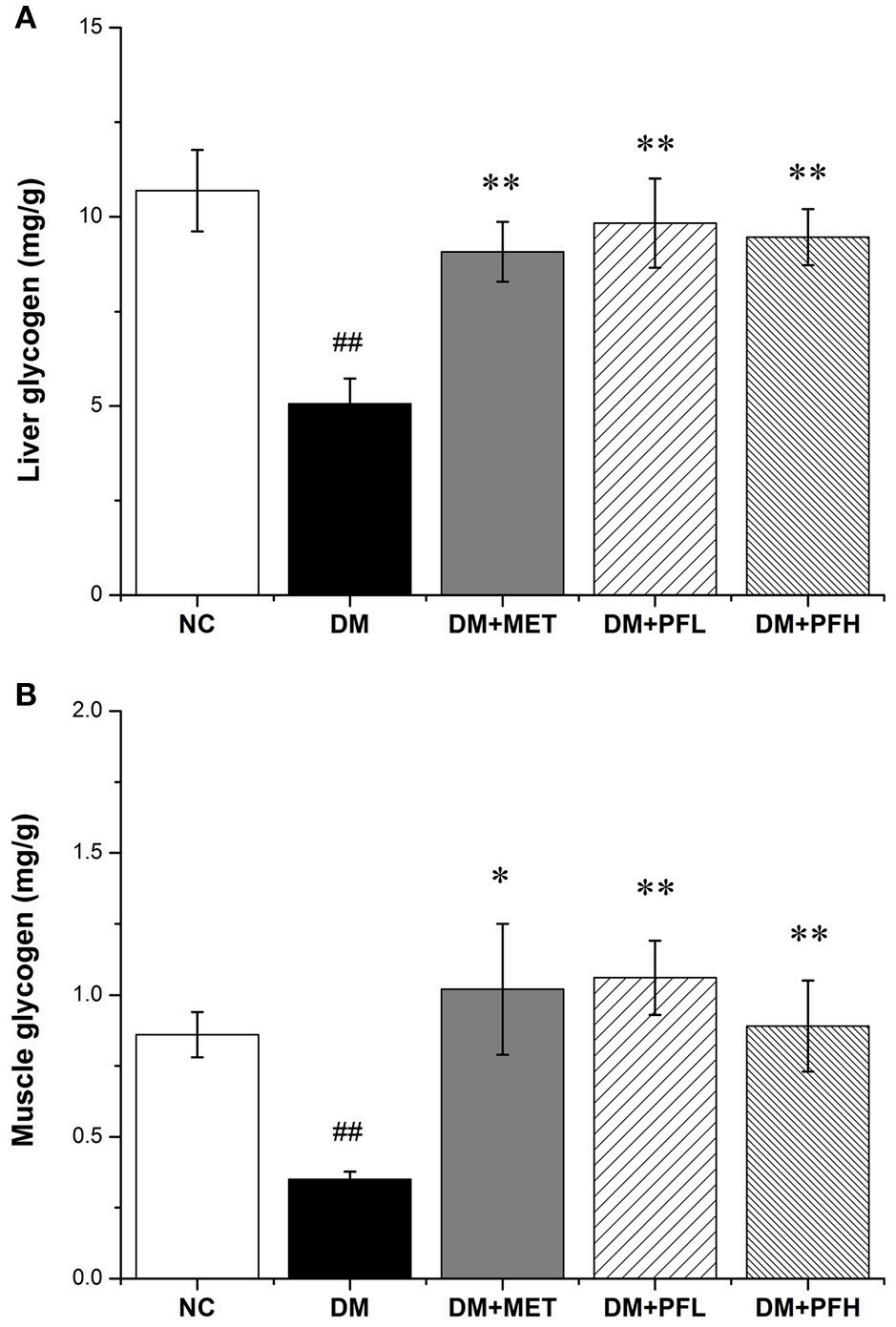
Effect of PGF on glycogen content. The glycogen content was assayed by a glycogen assay kit and expressed as mg/g tissue. **(A)** The glycogen storage in liver was promoted by PGF. **(B)** The glycogen content in muscle was increased by PGF. Data set shown is representative of three experiments. Data are represented as as mean ± *SD* (*n* = 10/group). ^##^*P* < 0.01, ^#^*P* < 0.05 vs. NC; ^**^P < 0.01, ^*^P < 0.05 vs. DM.

In order to test the effect of PGF on glucose metabolism, the content of liver glycogen was tested in DM rats. A reduction was observed in hepatic glycogen of diabetic rats. After the PGF treatment, the reduction was significantly attenuated by PFL (50%, ^**^p < 0.01) and PFH (48%, ^**^p < 0.01) (Figure 3A). The results suggest that PGF at low and high dosages effectively induced glycogen storage in liver. We also examined insulin sensitivity in the muscle tissue for glycogen content. The glycogen was increased in the PGF-treated groups compared with those of model group, suggesting an increase in insulin sensitivity in the muscle (Figure 3B).

Several antioxidant enzymes were examined in liver, which included catalase (CAT), superoxide dismutase (SOD) and glutathione peroxidase (GSH-Px). Those enzymes exhibited a reduction in DM group. The reduction was significantly improved by PGF and the improvement was similar to that of metformin (^*^p < 0.05 or ^**^p < 0.01) (Table 4). Malondialdehyde (MDA) level of DM group was significantly higher than the other groups (^#^*p* < 0.05). The increase of MDA in DM group was reduced by 30.4% (^*^p < 0.05) in the low-dose PGF group (PFL).

**Table 4:** Effect of PGF on antioxidant enzymes in rats liver (Mean ± *SD, n* = 10).

**Groups**	**CAT (U/mgprot)**	**SOD (U/mgprot)**	**GSH-PX (U/mgprot)**	**MDA(nmol/mgpro)**
NC	121.13 ± 6.01	249.43 ± 34.49	1590.55 ± 124.98	1.10 ± 0.26
DM	94.01 ± 6.75^#^	196.54 ± 6.45^#^	1097.08 ± 103.50^##^	2.76 ± 0.97^#^
DM+PFL	117.04 ± 7.14	279.09 ± 40.55	1208.77 ± 60.48^*^	1.92 ± 0.36^*^
DM+PFH	112.54 ± 6.47^*^	241.55 ± 21.85^*^	1405.21 ± 44.01^**^	2.47 ± 0.02
DM+MET	108.75 ± 10.67	246.84 ± 32.96	1389.96 ± 46.48^*^	2.35 ± 0.50

CAT, Catalase; SOD, Superoxide Dismutase; GSH-PX, Glutathione Peroxidase; MDA, Malondialdehyde. Data are expressed as mean ± SD (n = 10).

# p < 0.05,

## p < 0.01 for significant difference from the NC group.

* p < 0.05,

** *p < 0.01 for significant difference from the DM group. PFL: 50 mg/kg, PFH:100 mg/kg, MET: 150 mg/kg*.

### Western blotting analysis

**Figure 4:**
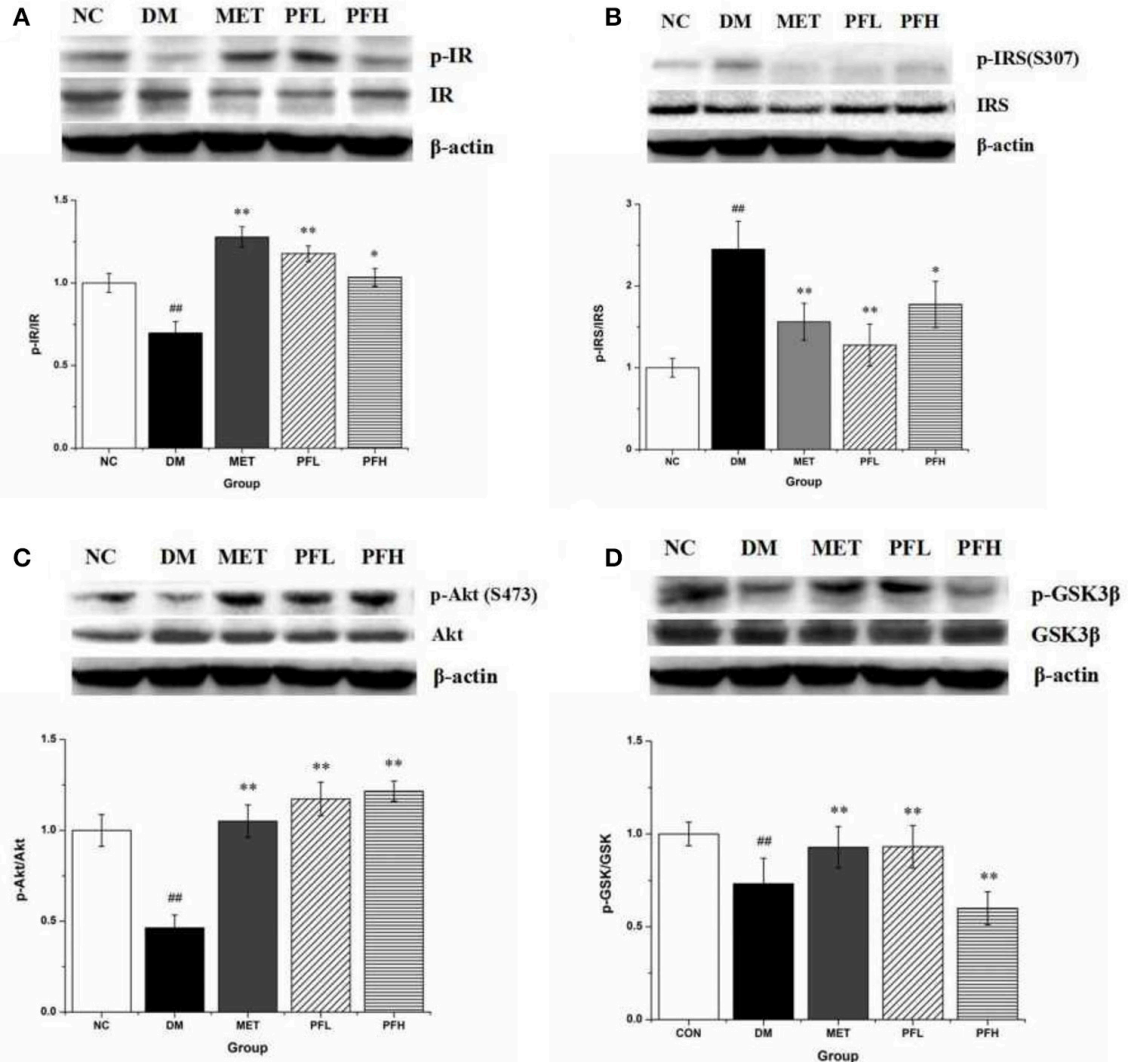
Effects of PGF on insulin signaling pathway. DM rats treated with PGF (50, 100 mg/kg) for 4 weeks. The protein expressions were analyzed by Western blot. **(A)** PGF increased the phosphorylation level of insulin receptor (IR) protein. **(B)** PGF decreased insulin receptor substrate (IRS) serine phosphorylation. **(C)** The phosphorylation of Akt protein was increased after PGF treatment. **(D)** The phosphorylated GSK-3β expression was promoted by PGF. β-actin is the same as shown in **(A-D)**. The results were normalized to β-actin. Data set shown is representative of three experiments. Data are represented as as mean ± *SD* (*n* = 10/group). ^##^*P* < 0.01, ^#^*P* < 0.05 vs. NC; ^**^P < 0.01, ^*^P < 0.05 vs. DM.

Insulin signaling was examined in liver tissue. Phosphorylation of IR, Akt and GSK-3β was significantly reduced and serine phosphorylation of IRS-1 was increased in DM rats (^##^*p* < 0.01) (Figures 4A–D), suggesting an impairment of insulin signaling in the liver of DM rats. These alterations were significantly improved by PFL and PFH (Figures 4A–D). These data suggest that the insulin signaling activity was improved by PGF in DM rats.

**Figure 5:**
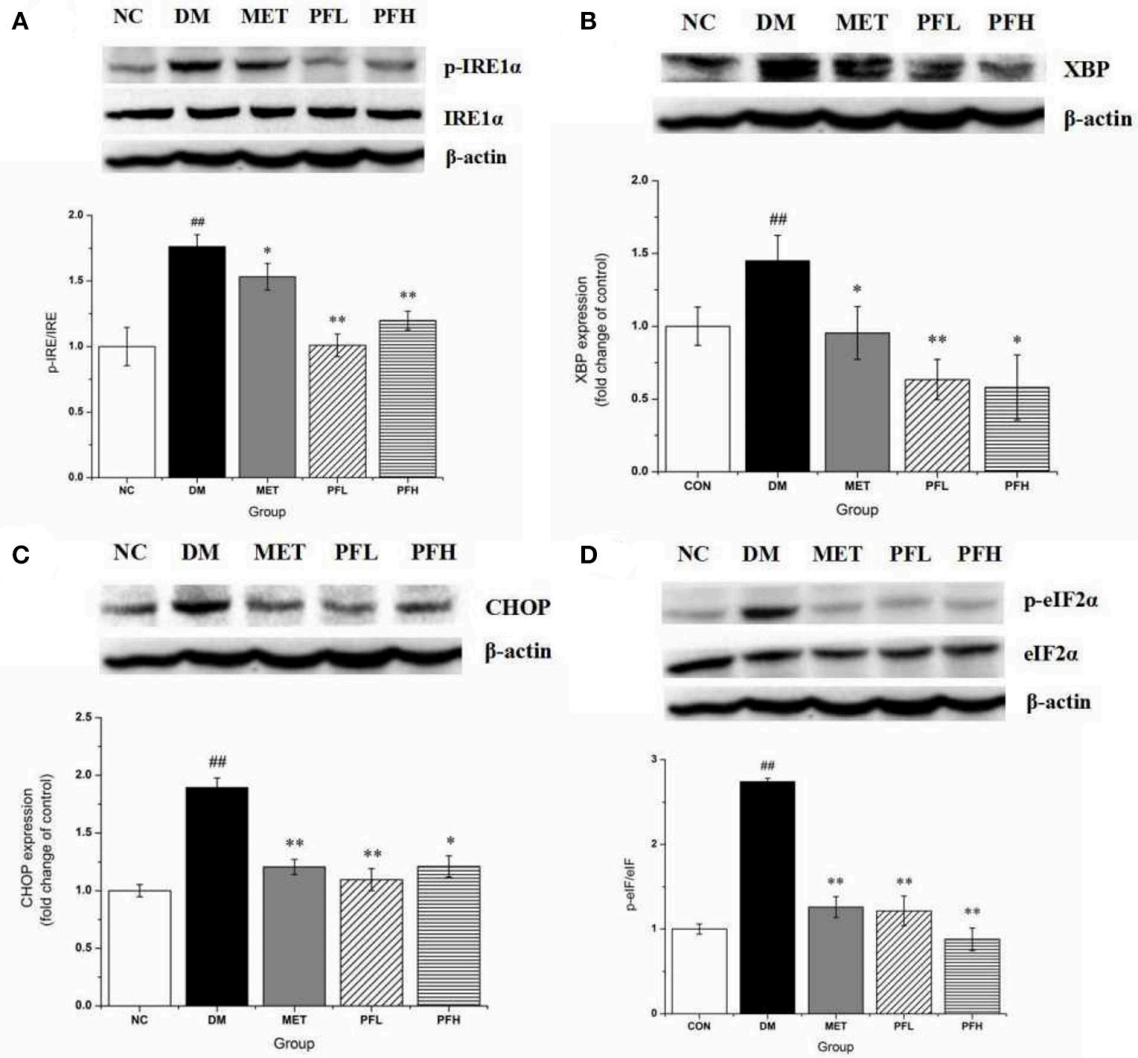
Effects of PGF on IRE1α-mediated URP pathway in liver tissue. DM rats treated with PGF (50, 100 mg/kg) for 4 weeks. **(A)** PGF decreased the expression of phosphorylated IRE1α. **(B)** The expression of XBP protein was reduced by PGF. **(C)** PGF decreased CHOP expression level. **(D)** PGF decreased the expression of phosphorylated eIF2α. β-actin is the same as shown in **(A–D)**.The results were normalized to β-actin. Data set shown is representative of three experiments. Data are represented as as mean ± *SD* (*n* = 10/group). ^##^*P* < 0.01, ^#^*P* < 0.05 vs. NC; ^**^P < 0.01, ^*^P < 0.05 vs. DM.

Endoplasmic reticulum (ER) stress is closely related to insulin resistance in obese mice (18). The effects of PGF on ER stress was examined by testing the phosphorylation of IRE1α and eIF2α, indicators of ER stress. The phosphorylation levels were significantly higher in DM rats (^##^*p* < 0.01). The increase was decreased by PFL and PFH (^**^p < 0.01) (Figures 5A,D). The expression of other ER stress signals (XBP1-s and CHOP) was enhanced in DM group (^##^*p* < 0.01). The increase was corrected by PGF treatment (Figures 5B,C). The results suggest that ER stress was significantly suppressed by PGF in DM rats.

### mRNA expression of ER stress genes

**Figure 6:**
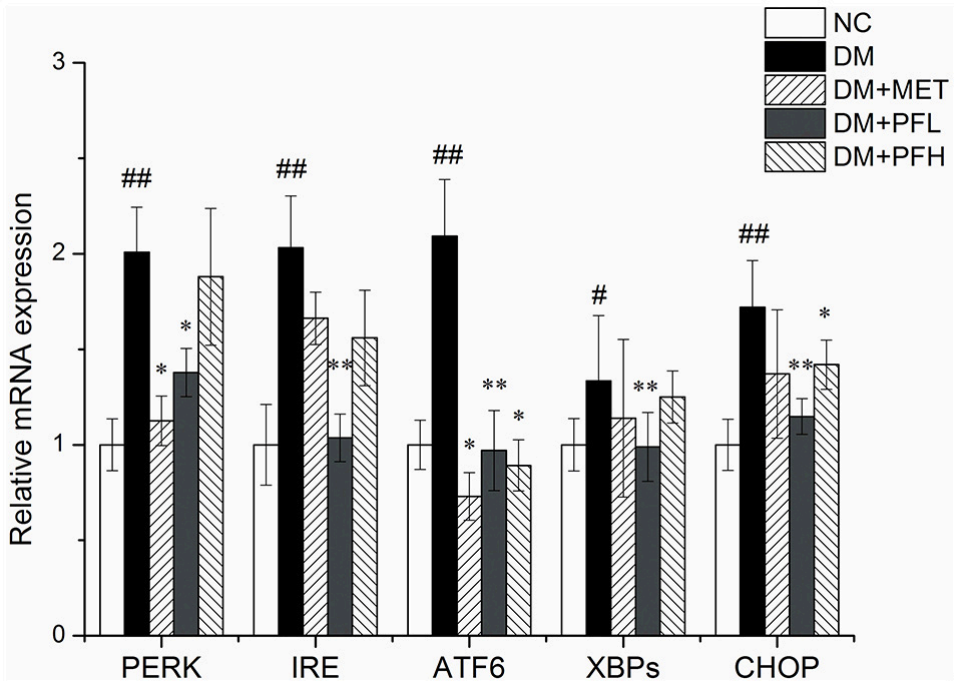
Effects of PGF on the relative mRNA expression of ER stress genes. DM group was intragastrically administered with PGF (50, 100 mg/kg) for 4 weeks. The relative levels of specific mRNAs were assessed by qRT-PCR. The gene expressions of PERK, IRE1α, ATF6, XBPs, and CHOP were increased in DM rats. The increase of these genes were reduced after treatment of PFL and PFH. The results were normalized to GAPDH. Data set shown is representative of three experiments. Data are represented as as mean ± *SD* (*n* = 10/group). ^##^*P* < 0.01, ^#^*P* < 0.05 vs. NC; ^**^P < 0.01, ^*^P < 0.05 vs. DM.

mRNA expressions of ER stress genes including PERK, IRE, ATF6, CHOP, and XBPs were examined in qRT-PCR. As shown in Figure 6, expression of these genes was increased in DM rats (^##^*p* < 0.01). The increase was significantly decreased by PFL and PFH(^**^p < 0.01 or ^*^p < 0.05). The efficacy of PGF was similar to that of metformin. There is no obvious difference between PGF and metformin groups.

## Discussion

Our data suggest that PGF may improve glucose metabolism through enhancement of insulin sensitivity. The type 2 diabetes rat model used in this study mimics the insulin resistance and hyperglycemia in T2DM patients (39, 40). Metformin was used as a positive control drug in the model for insulin sensitization. In the 4 weeks study, PGF treatment did not lead to the loss of appetite, suggesting that PGF has no significant anorexic effect on the rats at the dosages below 100 mg/kg. PGF treatment generated a reduction in blood glucose in the rats, which was observed with improvement of oral glucose tolerance test (OGTT), insulin tolerance test (ITT) and homoeostasis model assessment (HOMA-IR) index and insulin sensitivity index (ISI). In the positive control, metformin exhibited a similar activities. The data suggest that PGF is able to improve glucose metabolism through enhancement of insulin sensitivity. The efficacy is similar to that of metformin.

HOMA-IR, HOMA-β, and ISI were calculated according to fasting blood glucose and basal insulin values in the evaluation of the degree of peripheral insulin resistance and islet β-cell function (35). HOMA-IR was decreased, ISI and HOMA-β were increased by PGF, suggesting an improvement in insulin sensitivity and β-cell secretory function in the diabetic rats. In addition, glucose intolerance and impaired insulin tolerance are indicators of insulin resistance (41). These two parameters were both improved by PGF in the diabetic rats. The improvement is consistent with the change in HOMA-IR, HOMA-β

Insulin sensitivity in liver and muscle were improved by PGF. In this study, insulin resistance was observed with reduction of glycogen content and a reduction in insulin signaling activities. The glycogen content and insulin signaling activities were both enhanced by PGF. The signaling activities included the phosphorylation levels of IRS-1, Akt, and GSK-3β. The improvement of signaling activity may lead to the increase in glycogen content.

Fatty acids (FAs) play a critical role in the development of obesity-induced insulin resistance, a major risk factor for diabetes (42). Excess level of fatty acids may lead to insulin resistance through induction of mitochondrial activity for more ATP output, which will lead to inhibition of AMPK activity for an increase in ATP/AMP ratio (38). Furthermore, ATP may activate JNK to inhibit IRS-1 function in the insulin signaling pathway (43). PGF reduced the levels of TC, TG, and FFA in diabetic rats, indicating that it has a good effect of lowering blood lipids.

In addition, formation of free radicals was increased and antioxidant potential was decreased in the diabetic rats. The imbalance of oxidant/antioxidant defense systems was observed in alterations of antioxidant enzymes including SOD, CAT, GSH-PX, and impaired glutathione metabolism, which are common markers in the study of oxidative stress (44, 45). Our data suggest that PGF is able to reduce lipids and oxidative stress in the rat, which may contribute to the improvement of insulin sensitivity.

Endoplasmic reticulum stress (ERS) is associated with insulin resistance induced by HFD dietary. In this study, ERS was inhibited by PGF effectively for reduction of phospho-IRE1α and splicing of XBP-1 mRNA in the liver of DM rats. IRE1α is a bifunctional transmembrane kinase with both protein kinase and endoribonuclease activity for processing the splicing of the XBP1 mRNA. The results demonstrate that PGF may inhibit glucose-induced ERS in DM rats.

In this experiment, the low dose (PFL) had better effect than the high dose (PFH) in some parameters. This results may be due to the hormesis which is mentioned by Calabrese and Baldwin. The term “hormesis” refers to the phenomenon that chemicals, drugs, biological molecules or physical stressors exhibit opposing effects at low and high doses (46). The hormetic dose-response model has been documented in numerous biological, toxicological, and pharmacological investigations (47, 48). Many antibiotics, antiviral and anti-tumor agents, and numerous other medicines display hormetic-like biphasic dose responses: one dose may be effective clinically but another may be negative (49). Thus, PGF shows a beneficial effect on diabetic rats at low dose (PFL) but a less effect at high dose (PFH).

In conclusion, traditional herbs may be an excellent resource for natural products in the control of type 2 diabetes. In Uighur medicine, pomegranate flower is considered as a natural remedy for a number of diseases. Our data suggest that it has a strong activity in the control of T2DM. PGF extract was found to have a hypoglycemic effect through an increase in insulin sensitivity. The mechanism of regulation of insulin sensitivity involves in reduction of blood lipids and oxidative stress. On the other hand, our findings suggest that ERS is reduced by PGF. Pomegranate flower may be an excellent drug candidate for control of T2DM. In this study, the mechanism of PGF action was investigated in T2DM rats. The results suggest that PGF may act through inhibition of the ER stress pathway in the improvement of insulin sensitivity.

## Ethics statement

This study was carried out in accordance with the recommendations of the Animal Care Committee of Xinjiang Medical University of China. The protocol was approved by the Animal Care Committee of Xinjiang Medical University of China.

## Author contributions

DT participated in the experimental design, performed experiments, and wrote the paper. LL and DA conducted the research and analyzed the data. JY provided technical assistance and contributed in editing of the manuscript. JX provided material and technical assistance. HA and XX initiated the study and designed the setup of experiments. All authors read and approved the final manuscript.

### Conflict of interest statement

The authors declare that the research was conducted in the absence of any commercial or financial relationships that could be construed as a potential conflict of interest.
